# Novel mechanisms by which fat cells regulate systemic insulin sensitivity and diabetes risk

**DOI:** 10.1186/1753-6561-6-S3-O13

**Published:** 2012-06-01

**Authors:** Barbara B Kahn, Mark A Herman, Odile D Peroni

**Affiliations:** 1 Beth Israel Deaconess Medical Center and Harvard Medical School, USA

## 

The adipose cell functions as an endocrine organ in addition to its role in energy storage. Adipocytes secrete hormones, cytokines and other factors that influence energy balance, glucose homeostasis, insulin sensitivity and vascular biology through effects in peripheral tissues and the central nervous system. In humans with obesity and type 2 diabetes, expression of the Glut4 glucose transporter is down-regulated selectively in adipocytes and this has systemic metabolic effects. Knocking out Glut4 selectively in adipocytes in mice causes insulin-resistant and increases the risk of diabetes while adipose-specific Glut4 overexpression confers enhanced glucose tolerance and insulin sensitivity in spite of increased adiposity. To discover novel pathways that regulate glucose homeostasis and insulin sensitivity, we used DNA microarray analysis of adipose tissue from mice with adipose-specific alterations in Glut4 expression. We found novel adipocyte-secreted proteins that alter systemic insulin sensitivity. In addition, gene set enrichment analysis showed coordinate regulation of lipogenic genes in adipose tissue. This led to the discovery that the glucose-responsive transcription factor, Carbohydrate responsive-element binding protein (ChREBP), is a dominant regulator of lipogenesis in adipose tissue and that its expression in adipose tissue is highly associated with insulin sensitivity in humans even in the presence of obesity. Furthermore, we identified a novel, potent ChREBP isoform and defined a new “feed forward” mode of regulation of ChREBP by which adipose-lipogenesis regulates systemic glucose homeostasis. This could provide new strategies for prevention and treatment of insulin resistance and type 2 diabetes.

